# Quantitative assessment of lumbar spine bone marrow in patients with different severity of CKD by IDEAL-IQ magnetic resonance sequence

**DOI:** 10.3389/fendo.2022.980576

**Published:** 2022-09-20

**Authors:** Yan Xiong, Tongxiang He, Weiyin Vivian Liu, Yao Zhang, Shuang Hu, Donglin Wen, Yanan Wang, Peisen Zhang, Fan He, Xiaoming Li

**Affiliations:** ^1^ Department of Radiology, Tongji Hospital, Tongji Medical College, Huazhong University of Science and Technology, Wuhan, China; ^2^ Magnetic Resonance (MR) Research, GE Healthcare, Beijing, China; ^3^ Department of Nephrology, Tongji Hospital, Tongji Medical College, Huazhong University of Science and Technology, Wuhan, China; ^4^ College of Life Science and Technology, Beijing University of Chemical Technology, Beijing, China

**Keywords:** chronic kidney disease, bone marrow, IDEAL-IQ, PTH, R2*

## Abstract

**Background:**

Chronic kidney disease (CKD) has a significant negative impact on bone health. Bone marrow is an essential component of bone, mainly composed of trabecular bone and fat. The IDEAL-IQ sequence of MRI allows indirect quantification of trabecular bone mass by R2* and direct quantification of bone marrow fat content by FF map, respectively.

**Objective:**

Our objective was to explore the association of CKD severity with bone marrow using IDEAL-IQ and whether mineral and bone metabolism markers alter this association.

**Method:**

We recruited 68 CKD patients in this cross-sectional research (15 with CKD stages 3-4, 26 with stage 5, and 27 with stage 5d). All patients underwent lumbar spine IDEAL-IQ, BMD, and several bone metabolism markers (iPTH, 25-(OH)-VitD, calcium and phosphorus). Multiple linear regression analysis was used to examine the association of CKD severity with MRI measurements (R2* and FF).

**Results:**

More severe CKD was associated with a higher R2* value [CKD 5d versus 3-4: 30.077 s^-1^ (95% CI: 12.937, 47.217), *P* for trend < 0.001], and this association was attenuated when iPTH was introduced [CKD 5d versus 3-4: 19.660 s^-1^ (95% CI: 0.205, 39.114), *P* for trend = 0.042]. Furthermore, iPTH had an association with R2* value [iPTH (pg/mL): 0.033 s^-1^ (95% CI: 0.001, 0.064), *P* = 0.041]. Besides, FF was mainly affected by age and BMI, but not CKD.

**Conclusions:**

The bone marrow R2* value measured by IDEAL-IQ sequence is associated with CKD severity and iPTH. The R2* of IDEAL-IQ has the potential to reflect lumbar bone changes in patients with CKD.

## Introduction

Chronic kidney disease (CKD) affects 8-16% of the world’s population, with the global all-age prevalence growing by 29.3% from 1990 to 2017 (1, 2). CKD has a significant negative impact on bone health (3, 4). CKD-mineral and bone disorder (CKD-MBD) is the most common complication of CKD, a bone metabolic disease characterized by systemic bone, biochemical, and cardiovascular abnormalities that affect most patients from moderate to severe CKD (5, 6). Currently, clinicians can only roughly assess bone abnormalities in CKD patients based on clinical symptoms and commonly used clinical bone metabolism markers, including parathyroid hormone (PTH), vitamin D, phosphorus (P), and calcium (Ca) (7). This makes it important to find other clinically feasible methods to assess bone abnormalities in CKD.

Unlike primary osteoporosis (decrease in both trabecular and cortical bone), CKD patients always have secondary hyperparathyroidism, especially in end-stage patients (8). As PTH increases, trabecular and cortical bone behave differently (increases and decreases, respectively) (9, 10). In our previous study, we explored the changes of cortical porosity in patients with different stages of CKD (11). Trabecular bone (TB), which accounts for merely 20% of the total bone but two-thirds of the total bone surface area, shows greater metabolic activity than cortical bone (12). Moreover, TB is the main load-bearing bone of the vertebral body. Therefore, it is of great significance to study the changes of TB. Although it is challenging to obtain magnetic resonance imaging (MRI) signals of TB directly, it is possible to identify it indirectly. Studies have shown that bone marrow matrix in contact with TB exhibits an elevated transverse relaxation rate (R2*) because of local field inhomogeneities where mineralized matrix interfaced with it (13–15). The R2* value is approximately linearly related to TB density (16, 17), and increases as the interface area between TB and bone marrow matrix increases (13, 18). Therefore, R2* can indirectly quantify TB.

Besides TB, bone marrow fat (BMF) is an essential research topic of imaging studies on metabolic bone diseases since it is associated with the pathogenesis of bone loss (19). According to some research, BMF and TB density have a competitive relationship (20, 21). Only several studies have aimed at the association between CKD severity and BMF changes, but none of them included dialysis patients (22, 23). Dialysis is a key predictor of bone abnormalities in CKD patients (24), so it is essential to include them in the study.

MRI has been receiving widespread attention because of its non-invasive and non-radiation quantification of tissues. The iterative decomposition of water and fat with the echo asymmetry and least-squares estimation quantitation (IDEAL-IQ) sequence of MRI is a new water-fat separation algorithm developed from the IDEAL technology, which is a well-established clinical sequence with fast scanning time and no special post-processing. This sequence can generate fat fraction (FF) and R2* map in one scan (25, 26). Compared to traditional MRI techniques used to detect fat, this sequence further corrects common biases known in tissue fat measurement, including main magnetic field (B0) inhomogeneity, T1 effect, and T2* effect (27). It improves the water-fat separation from qualitative to quantitative. The FF map can directly measure the fat content in the tissue (i.e., liver and bone marrow) without further calculation (28, 29). The R2* map can also explain the inhomogeneity of the T2* effect/field, which is often used in liver iron assessment, such as liver iron overload and liver fibrosis (30, 31). Therefore, considering the imaging principle and the output results, this sequence shows excellent potential for investigating CKD bone marrow composition changes.

Besides, CKD patients are often combined with secondary hyperparathyroidism, vitamin D deficiency, and calcium and phosphorus metabolism disorders (5). And studies show that many bone metabolism markers, including PTH, are associated with abnormal cortical bone density, TB density, abnormal bone microstructure, and fracture (32).Therefore, we included several bone metabolism markers recommended by the KDIGO (Kidney Disease: Improving Global Outcomes) guidelines for the initial evaluation CKD-MBD (i.e., intact PTH (iPTH), 25-hydroxyvitamin D (25-(OH)-VitD), corrected calcium (cCa), phosphate (P)) and areal bone mineral density (aBMD) measured by dual-energy X-ray absorptiometry (DXA) (33, 34). Among them, PTH remains the best alternative biomarker for CKD-MBD (35). The aBMD is widely used in osteoporosis, but it is controversial in CKD, which deserves further study.

Our objective was to explore the association of CKD severity with bone marrow using IDEAL-IQ and whether mineral and bone biochemical parameters alter this association.

## Materials and methods

### Subjects

**Figure 1:**
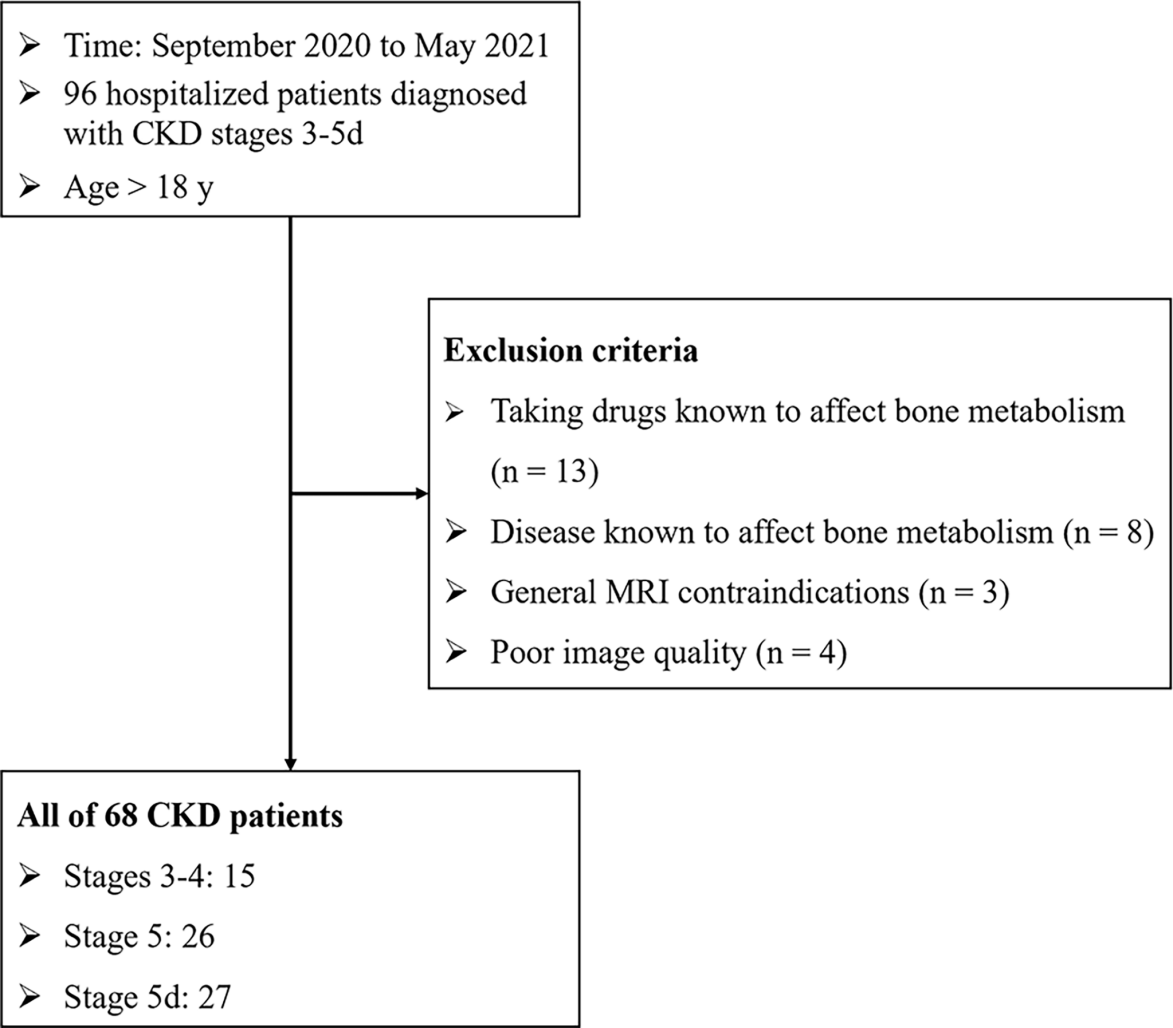
Flow chart of patient inclusion and exclusion.

The cross-sectional study was approved by the Medical Ethics Committee of Tongji Hospital, TJ-IRB20210108. Before the study, we obtained the written informed consent from all subjects. We registered the study on ClinicalTrials.gov as NCT04564924. Patients were recruited in the Department of Nephrology of Tongji Hospital from September 2020 to May 2021. All subjects were ambulatory and over 18 years old. The inclusion criteria were hospitalized patients diagnosed with CKD stages 3-5d. The exclusion criteria included taking drugs known to affect bone metabolism (e.g., steroid hormones, oral glucocorticoids, salmon calcitonin, and bisphosphonates); disease known to affect bone metabolism (e.g., hyperthyroidism, diabetes, rheumatic immunity disease, osteomalacia, rickets, scurvy, Paget’s disease, acromegaly, treatment with radiotherapy or chemotherapy, history of malignant tumors, fractures within six months, lumbar trauma surgery, motor neuron disease, scoliosis, and anorexia nervosa); and general MRI contraindications (e.g., cochlear implant, claustrophobia, pacemaker, and IUD). 68 patients were included in the final study population. Among them, 15 subjects were in CKD stages 3-4, 26 were in stage 5, and 27 were on maintenance hemodialysis (5d) at least three months. The flow chart of patient inclusion and exclusion was shown in **Figure 1**.

### MRI scanning

The study was carried out on a 3.0 T clinical scanner (Signa Pioneer, GE Healthcare, USA), the lumbar spine was scanned in a sagittal position using a spine coil while patients were placed in a supine position. Routine MRI sequences (T1 FSE, T2 FRFES, and T2 FLEX) were used to assess lumbar pathological findings, such as neoplastic lesions, compression fractures, lipomas, etc. Routine MRI parameters were provided in **Supplementary Material 1**. Besides routine sequences, the IDEAL-IQ sequence scan parameters were set as TE, minimum; TR, 8.4 ms; NEX, 2; Freq.FOV, 24 cm; flip angle, 4˚; slice thickness, 3 mm; in-plane spatial resolution, 1.5 mm × 1.5 mm; bandwidth, 83.33 kHz; and scan time, 2 minutes 24 seconds. FF and R2* maps were automatically generated after scanning.

### Vertebral bone marrow quantification

**Figure 2:**
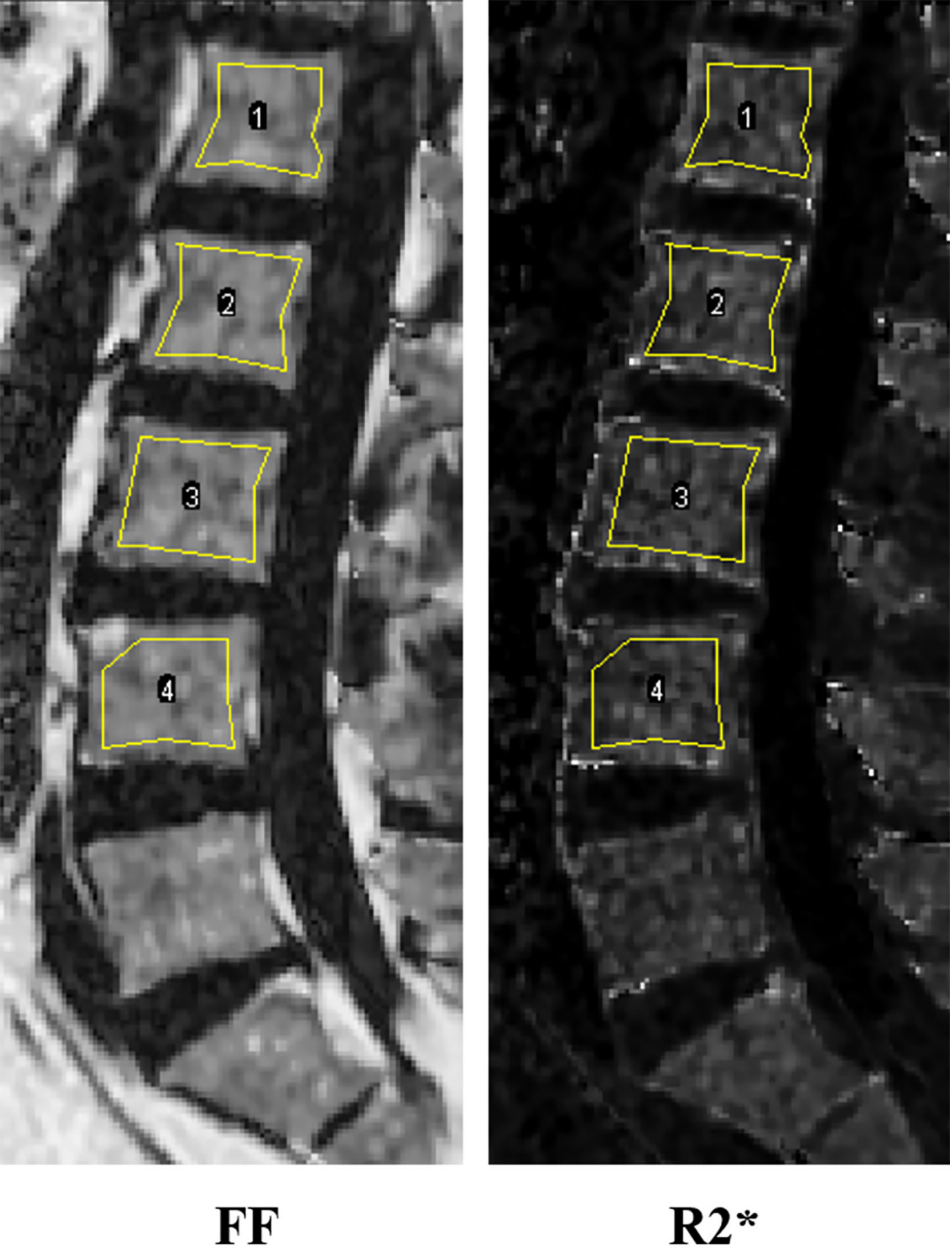
Representative IDEAL-IQ images and ROI of the lumbar spine (from L1 to L4).

The IDEAL-IQ imaging data (FF and R2* map) were analyzed using ImageJ (National Institutes of Health). All assessments were performed independently by two musculoskeletal radiologists with 3 and 5 years of experience, respectively, who were blinded to the clinical and DXA results. Similar to the lumbar DXA measurement, only the L1-L4 vertebrae were manually segmented. The ROIs were drawn on the mid-sagittal plane and the two para-mid-sagittal planes on the FF map and then copied to the R2* map. And the averages of all ROIs of FF and R2* were calculated respectively. ROIs were needed to avoid focal fatty degenerations, motion artifacts, the cortical bone of the vertebrae, vertebral discs, and the venous plexus. The ROI size could be adjusted based on the area of the vertebral body. **Figure 2** shows the example of ROIs.

### Laboratory analysis

Early morning fasting blood samples were drawn to evaluate serum markers. Laboratory tests were collected within one week before the MRI scan. Routine biochemical parameters including serum alkaline phosphatase (ALP), P, cCa, and creatinine (Cr) were determined using standard methods. Based on serum Cr, the estimated glomerular filtration rate (eGFR) was calculated through the CKD-EPI formula (36). The iPTH was measured on the Cobas e602 (Elecsys, Roche Diagnostics, Mannheim, Germany) using an electrochemiluminescence immunoassay (ECLIA). The 25-(OH)-VitD was analyzed by a chemiluminescence immunoassay on the Liaison XL (DiaSorin, Italy). The inter- and intra-assay coefficients of variation (CV) of iPTH and 25-(OH)-VitD were less than 4.31% and 7.87%, respectively. The minimum detection limit of iPTH and 25-(OH)-VitD were 1.20 pg/ml and 4 ng/ml, respectively. The normal ranges were as follows: iPTH, 15-65 pg/ml; 25-(OH)-VitD, lack < 12 ng/ml, insufficient 12-20 ng/ml, sufficient ≥ 20 ng/ml.

### Dual-energy X-ray absorptiometry

One week after MRI examination, the aBMD of the lumbar spine (from L1 to L4) was evaluated by DXA (Prodigy Lunar scanner, GE Healthcare, Waukesha, WI, USA).

### Statistical analysis

Data were presented as frequency (%) for categorical variables and mean ± standard deviation (SD) for continuous variables. The linear trends of baseline characteristics among three CKD groups (CKD stages 3-4, 5, and 5d) were acquired using Chi-squared statistics and one-way analysis of variance appropriately. Pearson’s and Spearman’s correlation analysis was performed to calculate the correlation between MRI measurements (FF and R2*) with demographics and other indicators respectively, according to Shapiro-Wilk Normality Test. The criteria for the Pearson *r* or Spearman *ρ*: higher than 0.8, strong correlation; 0.3-0.8, moderate correlation; lower than 0.3, weak correlation.

The association of CKD groups with MRI measurements was examined by multiple linear regression analysis. Firstly, an unadjusted model was established. Secondly, the model was adjusted for age, sex, and BMI. Finally, we added the significant indicators based on the correlation analysis mentioned above to the adjusted model. *P* for trends were calculated by treating CKD groups as ordered categorical variable (CKD 3-4 = 0, CKD 5 = 1, CKD 5d = 2). CKD groups were treated as unordered categorical variable in other linear regression analysis. The above three models were used to evaluate the association between CKD groups and MRI measurements, and whether adding indicators with significant correlations to the model could affect this association.

Finally, interobserver agreement between the two observers on parameter measurements was analyzed by calculating the interclass correlation coefficients (ICCs).

The *R* software (version 4.1.2) was performed for all statistical analyses. A two-tailed *P <*0.05 meant statistically significant.

## Results

### Baseline characteristics

Comparisons of demographics, bone metabolism markers, aBMD, and MRI measurements among CKD groups were presented in **Table 1**. More severe CKD patients had significantly higher BMI, P, iPTH, and R2* values. There was no significant difference in sex, age, ALP, cCa, aBMD, 25-(OH)-VitD, or FF.

**Table 1:** Baseline characteristics among three groups (CKD 3-4, 5, and 5d).

	Overall	CKD 3-4	CKD 5	CKD 5d	*P* for trend
Demographics					
Number	68	15 (22.1)	26 (38.2)	27 (39.7)	NA
Males	40 (58.8)	9 (60.0)	14 (53.8)	17 (63.0)	0.762
Age (years)	50 (13.2)	55.3 (11.4)	46.5 (13.9)	50.4 (12.8)	0.422
BMI (kg/m^2^)	22.7 (3.0)	24.0 (3.5)	22.7 (3.1)	21.8 (2.5)	**0.029**
eGFR (mL/min/1.73m^2^)	12.3 (11.7)	31.3 (10.9)	8.6 (3.4)	5.4 (2.3)	**<0.001**
ALP (U/L)	75 (29)	82 (33)	67 (24)	79 (29)	0.988
Bone metabolism markers					
P (mmol/L)	1.69 (0.70)	1.34 (0.37)	1.75 (0.73)	1.82 (0.76)	**0.047**
cCa (mmol/L)	2.23 (0.25)	2.29 (0.12)	2.19 (0.19)	2.24 (0.33)	0.666
iPTH (pg/mL)	269.4 (235.1)	108.3 (57.8)	199.6 (147.7)	426.0 (272.6)	**<0.001**
25-(OH)-VitD (ng/mL)	18.35 (9.39)	16.73 (9.53)	17.44 (9.27)	20.14 (9.48)	0.224
L1-L4 aBMD (g/cm^2^)	1.13 (0.17)	1.07 (0.14)	1.13 (0.18)	1.17 (0.17)	0.06
MRI measurements					
FF (%)	51.6 (11.6)	51.9 (9.6)	50.5 (12.7)	52.4 (11.8)	0.822
R2* (s^-1^)	155.2 (27.8)	140.7 (20.2)	149.7 (25.2)	168.5 (28.7)	**<0.001**

Categorical variables are summarized as count (%); continuous variables as mean (SD). *P* for trend reflect the significance of the linear trend across the CKD groups, using Chi-square and one-way ANOVA appropriately. Bold *P*-values consider statistical significance.

### Correlation analysis

FF only showed positive correlation with age (Spearman *ρ* = 0.373, *P* = 0.002) and BMI (Pearson *r* = 0.400, *P* < 0.001), while no correlation with other indicators. R2* was positively correlated with iPTH (Spearman *ρ* = 0.351, *P* = 0.003). There was no significant correlation between R2* with age, BMI and other indicators (**Table 2**).

**Table 2:** Correlation analysis of MRI measurements (FF and R2*) with demographics and clinical characteristics.

	FF (%)	*P*-value	R2* (s^-1^)	*P*-value
Age (years)	**0.373^#^**	**0.002**	-0.163^#^	0.183
BMI (kg/m^2^)	**0.400**	**<0.001**	0.035	0.776
ALP (U/L)	-0.021^#^	0.866	-0.169^#^	0.169
P (mmol/L)	-0.136^#^	0.269	0.228^#^	0.062
cCa (mmol/L)	0.051	0.677	-0.200	0.102
iPTH (pg/mL)	-0.025^#^	0.842	**0.351^#^**	**0.003**
25-(OH)-VitD (ng/mL)	-0.003^#^	0.978	0.166^#^	0.176
L1-L4 aBMD (g/cm^2^)	0.073	0.552	0.100	0.416

Data are presented as Pearson’s or Spearman’s rank (#) correlation coefficients appropriately. Bold *P*-values consider statistical significance.

### Linear regression analysis

The association of CKD groups with FF presented no statistical significance both in unadjusted and adjusted models, whereas two covariates presented significant effect in the adjusted model [age (year): 0.269% (95% confidence interval [CI]: 0.060%, 0.477%), *P* = 0.013; BMI (kg/m^2^): 1.371% (95% CI: 0.442%, 2.301%), *P* = 0.004] (**Table 3**).

**Table 3:** Association of CKD groups with FF (%) in unadjusted and adjusted models.

Independent variable	Unadjusted	Adjusted
CKD groups		
CKD 3-4	(ref.)	(ref.)
CKD 5	-1.366 (-8.977, 6.245)	2.433 (-4.401, 9.266)
CKD 5d	0.467 (-7.092, 8.026)	4.769 (-2.050, 11.588)
*P* for trend	0.822	0.158
Age (years)		**0.269 (0.060, 0.477)^a^**
BMI (kg/m^2^)		**1.371 (0.442, 2.301)^b^**
Sex		
Male		(ref.)
Female		3.665 (-1.461, 8.791)

Data are presented as FF% (95% CI). The adjusted model was adjusted for age, sex, and BMI.

Bold *P*-values consider statistical significance. ^a^ *P* < 0.05; ^b^ *P* < 0.01.

In the multiple linear regression models of CKD with R2*, CKD groups was positively associated with R2*, with a significant gradient in the unadjusted model [CKD 5d versus 3-4: 27.875 s^-1^ (95% CI: 11.331, 44.419), *P* for trend < 0.001]. After adjusting for age, sex, and BMI, the association remained [CKD 5d versus 3-4: 30.077 s^-1^ (95% CI: 12.937, 47.217), *P* for trend < 0.001]. Furthermore, no covariate presented a significant effect in the adjusted model (**Table 4**).

**Table 4:** Association of CKD groups with R2* (s^-1^) in unadjusted and adjusted models.

Independent variable	Unadjusted	Adjusted
CKD groups		
CKD 3-4	(ref.)	(ref.)
CKD 5	9.032 (-7.625, 25.690)	8.281 (-8.896, 25.458)
CKD 5d	**27.875 (11.331, 44.419)^a^**	**30.077 (12.937, 47.217)^b^**
*P* for trend	**<0.001**	**<0.001**
Age (years)		-0.365 (-0.890, 0.160)
BMI (kg/m^2^)		1.897 (-0.439, 4.232)
Sex		
Male		(ref.)
Female		-1.546 (-14.430, 11.339)

Data are presented as R2* in s^-1^ (95% CI). The adjusted model was adjusted for age, sex, and BMI.

Bold *P*-values consider statistical significance. ^a^ *P* < 0.05; ^b^ *P* < 0.01.

After introducing iPTH into the adjusted model, all the variance inflation factor (VIF) values were less than 5 suggesting that no multicollinearity existed. Interaction effects of CKD groups and iPTH were not statistically significant. We found that the association of CKD groups with R2* was attenuated but still significant [CKD 5d versus 3-4: 19.660 s^-1^ (95% CI: 0.205, 39.114), *P* for trend = 0.042]. At the same time, the regression coefficient of iPTH was statistically significant [iPTH (pg/mL): 0.033 s^-1^ (95% CI: 0.001, 0.064), *P* = 0.041], suggesting that iPTH was still associated with R2* after adjusted age, sex, BMI, and CKD groups (**Table 5**).

**Table 5:** Association of R2* (s^-1^) with CKD groups and iPTH in the adjusted model.

Independent variable	Adjusted + iPTH
CKD groups	
CKD 3-4	(ref.)
CKD 5	5.522 (-11.421, 22.465)
CKD 5d	**19.660 (0.205, 39.114)^a^**
*P* for trend	0.042
iPTH (pg/mL)	**0.033 (0.001, 0.064)^a^**

Data are presented as R2* in s^-1^ (95% CI), adjusted for age, sex, and BMI.

Bold *P*-values consider statistical significance. ^a^ *P* < 0.05.

### Interobserver agreement

The ICCs for R2* and FF was 0.965 (95% CI: 0.944 -0.978) and 0.958 (95% CI: 0.933-0.974), respectively.

## Discussion

We investigated the association between CKD severity and lumbar bone marrow FF and R2* values. We found R2* was associated with CKD and iPTH, independent of age or BMI. In contrast, FF was mainly affected by age and BMI, but not CKD in our study. Despite the growing recognition of the importance of bone marrow composition in bone biomechanics, there are still few studies on bone marrow in CKD patients.

The underlying mechanism of the association between CKD severity and BMF content is not fully understood. We found that although FF was little affected by CKD severity, it was significantly affected by age and BMI, which was consistent with other studies. Veldhuis et al. (19) found a constant increase in BMF with age in healthy adults. Cohen et al. (37) found that obese individuals had a higher rate of bone marrow obesity compared with overweight and healthy subjects measured with bone biopsy. Similar results were found in older and young men, as well as post- and pre-menopausal women (38–41). This reminds us that when studying the changes in BMF caused by metabolic diseases, we need to strictly control the effects of age and BMI to avoid getting wrong conclusions. In the few two imaging studies on lumbar BMF in patients with CKD (22, 23), a small sample study found that vertebral BMF in CKD stages 3b-4 (n=8) was 13.8% higher than healthy controls (n=8); another study found that vertebral BMF in eGFR < 45 mL/min/1.73m^2^ (n=58) was 3.7% higher than eGFR > 60 mL/min/1.73m^2^ (n=297). Both studies suggest that patients with CKD tended to have increased BMF compared to healthy controls. However, among the CKD patients in our study, BMF did not increase significantly when CKD severity increased. Probably because they compared CKD patients with healthy people, whereas our study subjects were all CKD patients, the cohort structure was different. Therefore, in patients with severe CKD, BMF may have reached a plateau and will not increase significantly with disease progression.

In this study, we found that bone marrow R2* value was higher in more severe CKD, and adjustment of iPTH attenuated the original association between CKD groups and R2*. This means that changes in R2* are associated with both CKD and iPTH. This may be related to end-stage CKD trabecular sclerosis. Trabecular sclerosis has long been regarded as a feature of MBD, and is more pronounced in patients with uremia. Although this increase does not imply an increase in bone strength, as irregular TB may lose its appropriate connectivity and 3D structure (42, 43). Meanwhile, there is strong indirect evidence that secondary hyperparathyroidism is a major cause, as this condition always occurs with CKD progression (8, 9). This may be related to the anabolic action of PTH on TB (8). The best explanation for PTH to induce bone anabolism in TB is that it promotes osteoblast survival and/or osteoblastogenesis (44). In the CKD, bone is regarded as one of the classical targets of PTH because PTH1R (PTH 1 receptor) is expressed in osteoclasts, osteocytes, and osteoblasts (45). PTH excess usually has an anabolic action on TB and a catabolic action on cortical bone. The reasons for this differential effect of PTH on trabecular and cortical bone are not fully understood (9, 44). However, since adjusting iPTH only attenuated the association but did not make it disappear, suggesting that there were other factors in CKD that impair bone health. Previous studies have shown that abnormalities such as chronic metabolic acidosis and chronic inflammation caused by CKD can also impair bone health (46).

There was no significant difference in aBMD among the three groups and no significant correlation between aBMD and R2*. We consider the main reason is that the aBMD is the projection of a 3D structure on a 2D plane, so it can’t distinguish between cortical and trabecular bone (47). MBD is strongly influenced by PTH. As PTH increases, trabecular and cortical bone behave differently (increases and decreases, respectively) (9, 10). In short, the increased trabecular BMD can mask the reduced cortical BMD, thus giving an inconsistent result with the actual bone disease. Therefore, the lack of correlation between the aBMD and R2* values of the lumbar spine may reflect the technical limitations of DXA, not a lack of correlation between the true trabecular BMD and R2* values.

Besides, 25-(OH)-VitD did not tend to decrease with more severe CKD stages. The possible reason for this is that most patients were supplemented with vitamin D. Studies reveal that exogenous vitamin D supplementation can increase 25-(OH)-VitD (24). Therefore, it is limited for 25-(OH)-VitD to evaluate bone metabolism in CKD.

The advantages of this study include: (1) IDEAL-IQ is a sequence that has already been used in clinical applications and provides convenient quantification of bone marrow components; (2) This study included more comprehensive bone metabolism markers at one time; (3) The exclusion criteria were strictly established in this study to exclude patients related to diseases or drugs that may affect bone metabolism, making results more reliable. However, this study also has some limitations: (1) This study is exploratory cross-sectional and cannot determine a causal association between CKD severity and bone marrow, therefore more prospective studies are required; (2) We didn’t include many other factors that affect bone, such as chronic inflammation, chronic metabolic acidosis, and premature hypogonadism.

In conclusion, the bone marrow R2* value measured by IDEAL-IQ sequence is associated with CKD severity and iPTH. The R2* of IDEAL-IQ has the potential to reflect lumbar bone changes in patients with CKD.

## Data availability statement

The raw data supporting the conclusions of this article will be made available by the authors, without undue reservation.

## Ethics statement

The studies involving human participants were reviewed and approved by Medical Ethics Committee of Tongji Hospital, Tongji Medical College, Huazhong University of Science and Technology. The patients/participants provided their written informed consent to participate in this study.

## Author contributions

FH and XL concept and design. YX and TH designed the study, evaluated the data, and wrote the manuscript. DW devised the outline of the manuscript. YW, SH, and YZ collected the information and analyzed the data. PZ created the figures for the manuscript. WL and XL critically revised the manuscript. All authors contributed to the article and approved the submitted version.

## Funding

This study was supported by the National Natural Science Foundation of China (NSFC) (No. 81930045 and 31630025).

## Conflict of interest

WL was employed by GE Healthcare.

The remaining authors declare that the research was conducted in the absence of any commercial or financial relationships that could be construed as a potential conflict of interest.

## Publisher’s note

All claims expressed in this article are solely those of the authors and do not necessarily represent those of their affiliated organizations, or those of the publisher, the editors and the reviewers. Any product that may be evaluated in this article, or claim that may be made by its manufacturer, is not guaranteed or endorsed by the publisher.
