# Supplementary material for: Quantitative assessment of lumbar spine bone marrow in patients with different severity of CKD by IDEAL-IQ magnetic resonance sequence
Source: Front Endocrinol (Lausanne). 2022 Sep 20;13:980576. doi: 10.3389/fendo.2022.980576 (PMC9530399; doi:10.3389/fendo.2022.980576)
Supplement: Supplementary file 1 [file Table_1.docx]

The scanning parameters for conventional sequences are as follows:

(a) Sagittal T2 FRFSE: TE, 102 ms; TR, 2000 ms; NEX, 2; Freq.FOV, 32 cm; slice thickness, 4 mm; in-plane spatial resolution, 0.8 mm × 1.0 mm; bandwidth, 62.50 kHz; and scan time, 1 minutes 8 seconds.

(b) Sagittal T2 FLEX: TE, 85.0 ms; TR, 1864 ms; NEX, 2; Freq.FOV, 32 cm; slice thickness, 4 mm; in-plane spatial resolution, 1.0 mm × 1.4 mm; bandwidth, 50.00 kHz; and scan time, 1 minutes 52 seconds.

(c) Sagittal T1 FSE: TE, Min Full ms; TR, 577 ms; NEX, 2; Freq.FOV, 32 cm; slice thickness, 4 mm; in-plane spatial resolution, 0.9 mm × 1.2 mm; bandwidth, 62.50 kHz; and scan time, 1 minutes 5 seconds.
